# Sensor Technologies for Caring People with Disabilities

**DOI:** 10.3390/s19224914

**Published:** 2019-11-11

**Authors:** Francisco José García-Peñalvo, Manuel Franco-Martín

**Affiliations:** 1 GRIAL Research Group, Computer Science Department, Research Institute for Educational Sciences, University of Salamanca, Paseo de Canelejas 169, 37008 Salamanca, Spain; 2 Department of Psychiatry, Zamora Hospital, 49022 Zamora, Spain; mfranco@usal.es; 3 Department of Psychiatry, Hospital Universitario Rio Hortega, 47012 Valladolid, Spain

**Keywords:** technological ecosystems, eHealth, people with disabilities, assisted living, privacy, artificial intelligence

## Abstract

Today, the population uses technology for every daily activity involving business, education, communication, entertainment, etc. Technology may also help us to take care of people who suffer some kind of disability. Complex technological ecosystems with pervasive and intelligent capabilities get along with us, facilitating the vigilance of those who need special attention or assisted living cares due to their health limitations. The advances in sensor research have enriched the powerful of these ecosystems to achieve more sophisticated monitoring and alarm systems, also taking into account the balance between the level of assistance and the people’s privacy. The Special Issue on “Sensor Technologies for Caring People with Disabilities” aims to present recent developments on sensor technologies for caring people with disabilities, focusing on the different configurations that can be used and novel applications in the field.

## 1. Introduction

Today, it is a fact that the world is ageing. A characteristic feature of older people is the frequent occurrence of both cognitive and physical impairments. In the European Union, approximately 17.8% of the population was aged 65 or over at the beginning of 2012. This fact implies an increase in the cost of care for this population. Globally, 46.8 million people live with dementia, and this number is expected to rise alarmingly to 131.5 million by 2050 [1]. According to the World Health Organization [2], over a billion people, about 15% of the world’s population, have some form of disability. Furthermore, the rapid growth of the ageing population is causing an increase in chronic health conditions, and therefore a rise in the population rates of disability. Additionally, people with disabilities have less access to health care services and are more prone to experiencing unmet health care needs.

Most dependent older people live in their homes and are cared for not only by their spouse or other family member but also by neighbours or friends who play the role of informal carer and are not paid for it. These informal carers often provide adequate and complete care for their continuing changes and extreme situations that require their care, increasing the demand for care as they progress through the different stages of deterioration.

On the other hand, the proportion of people aged 65 and over living in rural areas is very high. This situation poses new challenges in how to improve the independence and quality of life of these people and their caregivers.

Technology provides solutions that can be useful to help in these situations [3,4,5,6]. In recent years, information systems responsible for supporting information and knowledge management in heterogeneous contexts have evolved into what is now called technological ecosystems [7,8]. The definition of technological ecosystem varies from one author to another, but all agree on one fundamental point: there is a clear relationship between the characteristics of a natural ecosystem and a technological ecosystem in any of its variants [9,10,11,12]. In this way, the technological ecosystem can be defined as a set of software components that relate to each other using information flows in a physical medium that provides the support for these flows [13] and also takes into account the system users as another component of the ecosystem [14].

In this sense, recent advances in sensor research and innovation have boosted the prospects of the use of these technologies for assisting people with disabilities [15,16]. Sensors are used for many different purposes in regard to disabled people [17]. Monitoring and alarm systems, for example, can ameliorate the adverse effects of unpredictable events, such as sudden illness, falls, or wandering [18,19]. Pressure sensors have been employed in robotics for the treatment of children with autism [20,21]. Inertial Measurement Units (IMUs) and laser systems have been used for helping blind people [22,23], for example building virtual canes [24]. In sum, the use of sensors can improve the quality of life of people with disabilities, as well as promoting their independence.

This Special Issue has converged at collecting high-quality papers aimed at solving well-known technical problems and challenges typical of caring people with disabilities using sensor-based technologies. The primary purpose has been to combine innovative proposals efficiently, converging on the performance evaluation and the comparison with existing approaches. The Guest Editors picked eleven high-level contributions for publication after several rounds of reviews carried out by invited experts.

## 2. A Review of the Contributions in this Special Issue

The Special Issue has two papers oriented to the state-of-the-art and nine research papers.

In the first review paper, the authors analysed thirty-seven papers (2003–2018) related to the application of technological ecosystems to the care and assistance domain [25]. The main findings show that it is indeed an emerging field; few of the found ecosystem proposals have been developed in the real world nor have they been tested with real users. In addition, much research to date reports the proposal of platform-centric architectures developed over existing platforms not developed explicitly for care and services provided. Employed sensor technologies for providing services have very diverse natures depending on the intended services to be provided. However, many of these technologies do not take into account medical standards. With this information, the authors had enough data to model an eHealth technological ecosystem for caregivers [26,27].

The second state-of-the-art paper is devoted to providing an updated, holistic view of navigation devices capable of guiding the blind through indoor and/or outdoor scenarios, in order to enable developers to exploit the different aspects of its multidisciplinary nature [28].

A first group of research papers is related to website adjustments and recommendations for people with different kinds of disabilities. Alonso-Virgós et al. stated that users with Down syndrome suffer problems in web browsing due to website accessibility barriers related to their cognitive disability. Thus, they proposed a guide that extracts a list of useful accessibility and usability guidelines for web developers based on a neurological study of Down syndrome [29]. On the other hand, Kous and Polančič [30] investigated how people with dyslexia respond to a customised version of a website in terms of its effectiveness, efficiency, satisfaction and suitability when compared to the default version of the website. They customised a website with the aid of integrated assistive technology that offers people with dyslexia the opportunity to adjust a website themselves by their individual needs, demands and preferences. Therefore, the primary contributions of this research are the empirical insights of interaction with both the default and customised version of the website for people with dyslexia.

Two research papers are related to blind disability. Lin et al. introduced Visual Localizer, which is composed of ConvNet descriptor and global optimisation, to achieve robust visual localisation for assisted navigation. To further improve the robustness of image matching, the authors utilised the network flow model as a global optimisation of image matching [31]. Márquez-Olivera et al. developed an artificial intelligence-based system which recognises faces in real-time for people with visual impairment or prosopagnosia who have limitations when trying to identify people visually [32].

Three research papers have a great relationship with the human–computer interaction area. Moreno et al. presented a portable platform for training in ultrasound imaging-based musculoskeletal exploration in rehabilitation settings using an IMU sensor based on micro-electromechanical systems. Ultrasound imaging in the diagnostic and treatment of musculoskeletal pathologies offers various advantages, but it is a strongly operator-dependent technique, so training and experience become of fundamental relevance for rehabilitation specialists. The critical element of the proposed platform is a replica of a real transducer (HUSP—Haptic US Probe), equipped with micro-electromechanical systems based IMU sensors, an embedded computing board to calculate its 3D orientation and a mouse board to obtain its relative position in the 2D plane [33]. Ramírez-Martínez et al. tackled the problem of electromyography signal classification, solved with the proposed signal processing and feature extraction stages, with the focus lying on the signal model and time domain characteristics for better classification accuracy. The proposal considers a simple preprocessing technique that produces signals suitable for feature extraction and the Burg reflection coefficients to form learning and classification patterns [34]. Torres-Carrión et al. focused on the reduction in cognitive abilities caused by the Down syndrome, with visual-motor skills being particularly affected. They proposed stimulating the cognitive visual-motor skills of individuals with Down syndrome using exercises with a gestural interaction platform based on the KINECT sensor named TANGO:H, the goal being to improve them [35].

The last two papers apply different kinds of sensors for helping people with Parkinson’s disease and autism. Albani et al. proposed a system suitable for the remote monitoring of Parkinson’s disease subjects. It consists of the integration of two approaches: low-cost optical devices for the upper limbs and wearable sensors for the lower ones. The system performs the automated assessments of six motor tasks of the unified Parkinson’s disease rating scale, and it is equipped with a gesture-based human–machine interface designed to facilitate the user interaction and system management [36]. Tomczak et al. presented the application of a bluetooth skin resistance sensor in assisting people with autism spectrum disorders in their day-to-day work. They considered the best placement of the sensor, on the body, to gain the most accurate readings of user stress levels, under various conditions. They also determined the placement of the sensor based on wearer convenience [37].
